# Genome Sequencing Reveals the Origin of the Allotetraploid *Arabidopsis suecica*

**DOI:** 10.1093/molbev/msw299

**Published:** 2017-01-12

**Authors:** Polina Yu. Novikova, Takashi Tsuchimatsu, Samson Simon, Viktoria Nizhynska, Viktor Voronin, Robin Burns, Olga M. Fedorenko, Svante Holm, Torbjörn Säll, Elisa Prat, William Marande, Vincent Castric, Magnus Nordborg

**Affiliations:** 1 Gregor Mendel Institute, Austrian Academy of Sciences, Vienna Biocenter (VBC), Vienna, Austria; 2 Vienna Graduate School of Population Genetics, Institut für Populationsgenetik, Vetmeduni, Vienna, Austria; 3 Université de Lille CNRS, UMR 8198 - Evo-Eco-Paleo, Villeneuve d'Ascq, France; 4 Institute of Biology, Karelian Research Center of the Russian Academy of Sciences, Republic of Karelia, Petrozavodsk, Russia; 5 Faculty of Science, Technology and Media, Department of Natural Sciences, Mid Sweden University, Sundsvall, Sweden; 6 Department of Biology, Lund University, Lund, Sweden; 7 Centre National de Ressources Génomiques Végétales, INRA-CNRGV, Castanet-Tolosan, France

**Keywords:** polyploidy, *Arabidopsis suecica*, *Arabidopsis thaliana*, *Arabidopsis arenosa*, shared polymorphism, speciation, hybridization

## Abstract

Polyploidy is an example of instantaneous speciation when it involves the formation of a new cytotype that is incompatible with the parental species. Because new polyploid individuals are likely to be rare, establishment of a new species is unlikely unless polyploids are able to reproduce through self-fertilization (selfing), or asexually. Conversely, selfing (or asexuality) makes it possible for polyploid species to originate from a single individual—a *bona fide* speciation event. The extent to which this happens is not known. Here, we consider the origin of *Arabidopsis suecica*, a selfing allopolyploid between *Arabidopsis thaliana* and *Arabidopsis arenosa*, which has hitherto been considered to be an example of a unique origin. Based on whole-genome re-sequencing of 15 natural *A. suecica* accessions, we identify ubiquitous shared polymorphism with the parental species, and hence conclusively reject a unique origin in favor of multiple founding individuals. We further estimate that the species originated after the last glacial maximum in Eastern Europe or central Eurasia (rather than Sweden, as the name might suggest). Finally, annotation of the self-incompatibility loci in *A. suecica* revealed that both loci carry non-functional alleles. The locus inherited from the selfing *A. thaliana* is fixed for an ancestral non-functional allele, whereas the locus inherited from the outcrossing *A. arenosa* is fixed for a novel loss-of-function allele. Furthermore, the allele inherited from *A. thaliana* is predicted to transcriptionally silence the allele inherited from *A. arenosa*, suggesting that loss of self-incompatibility may have been instantaneous.

## Introduction

Polyploidy requires a series of unlikely events: the formation of unreduced gametes, hybridization, and the establishment of a new polyploid population (Ramsey and Schemske 1998; Soltis et al. 2015). Nevertheless, whole-genome duplication events have occurred throughout evolutionary history, and have been frequent in plants (Vision et al. 2000; Jiao et al. 2011; Vanneste, Baele, et al. 2014).

The genus *Arabidopsis* includes two relatively young allotetraploid species: *Arabidopsiskamchatica* and *Arabidopsissuecica* (Hylander 1957; Shimizu et al. 2005; Shimizu-Inatsugi et al. 2009). The former is a hybrid between *A. lyrata* and *A. halleri* and is limited to East Asia and North America (Shimizu et al. 2005); the latter is a hybrid between *A. thaliana* and *A. arenosa* and is limited to the Fennoscandinavian region (O’Kane et al. 1996). Previous studies have suggested that *A. suecica* originated from a single hybridization event between 12 and 300 Kya (Jakobsson et al. 2006) with *A. thaliana* as the maternal parent (Price et al. 1994; Hurka 1995; Comai et al. 2000; Säll et al. 2003). The latter conclusion is based partly on sequences from maternally inherited chloroplast genomes, partly on the fact that “synthetic” allotetraploids can be generated by fertilizing autotetraploid *A. thaliana* (which occur rarely in nature, but can readily be generated in the laboratory) with pollen from naturally autotetraploid *A. arenosa* (which are common), whereas the reciprocal cross cannot be made (Comai et al. 2000). Thus, the most likely scenario for the formation of *A. suecica* is that in which a normal (diploid) pollen from tetraploid *A. arenosa* fertilizes an unreduced gamete of diploid *A. thaliana* (Jakobsson et al. 2006). In support of this scenario, the *A. arenosa* complement of *A. suecica* is more closely related to tetraploid rather than diploid *A. arenosa* (Novikova et al. 2016). The alternative scenario of mating between diploid parents followed by whole-genome duplication seems less likely.

Whether inheritance in an allotetraploid will be disomic or tetrasomic (or meiosis will fail and lead to aneuploidy) depends largely on the divergence between the parental species (because it allows to prevent homologous pairing), but can also be controlled by specific molecular mechanisms (Griffiths et al. 2006). Cytological studies revealed a diploid-like, homologous chromosomal pairing in *A. suecica* (Comai et al. 2003). Interestingly, synthetic lines appear to be much less stable (Comai et al. 2000; Madlung et al. 2005; Henry et al. 2014). It has been suggested that such meiotic regularity has a genetic basis and is under selection in polyploids (Henry et al. 2014).

*Arabidopsis* *suecica* is currently widely used as a model for studying allotetraploidy in terms of the evolutionary retention of homologs (Chang et al. 2010), the epigenetic regulation of nucleolar dominance (Chen et al. 1998; Pikaard 1999; Pontes et al. 2007; Costa-Nunes et al. 2010; Pontvianne et al. 2012), overall gene expression (Wang et al. 2006; Ha et al. 2009; Ng, Miller, et al. 2014; Ng, Shi, et al. 2014; Tian et al. 2014; Miller et al. 2015), and heterosis (Solhaug et al. 2016). One of the main advantages of *A. suecica* as a model (in addition to the fact that one of the parents is the model plant *A. thaliana*), is the possibility to “re-run evolution” by creating synthetic hybrids (Chen et al. 1998; Comai et al. 2000). However, to fully capitalize on this, it is important to understand the history and origin of the natural species better: hence this article. Using whole genome sequencing data of multiple natural *A. suecica* accessions that cover most of its geographic distribution, we aim to describe the population history of this allotetraploid species: the location and timing of its origin and also the evolution of its ability to self-fertilize which ultimately led to the establishment of *A. suecica* as a new species.

## Results and Discussion

We sequenced (using Illumina 100-bp paired-end reads) 15 natural accessions of *A. suecica* sampled at different locations throughout the species distribution (supplementary table S1 and fig. S1, Supplementary Material online). We mapped *A. suecica* reads to the *A. thaliana* and *A. lyrata* reference genomes simultaneously, and obtained variant calls from the *A. thaliana* and *A. arenosa* components of *A. suecica*, respectively (see “Materials and Methods” section). Our approach was greatly facilitated by the fact that *A. suecica* accessions are natural inbred lines as a result of selfing: by only retaining homozygous calls, we avoid many spurious polymorphisms that would have arisen from the misalignment of reads to the wrong parental genome. We mapped 77% of the raw reads on average (supplementary table S1, Supplementary Material online), identifying 167,283 polymorphic sites in 15 *A. suecica* accessions on the *A. thaliana* portion of the reference and 416,898 sites on the *A. lyrata* portion. Throughout the study, results generated for *A. suecica* are compared with data from the parental species *A. thaliana* (1001 Genomes Consortium 2016) and *A. arenosa* (Novikova et al. 2016).

**Fig. 1:**
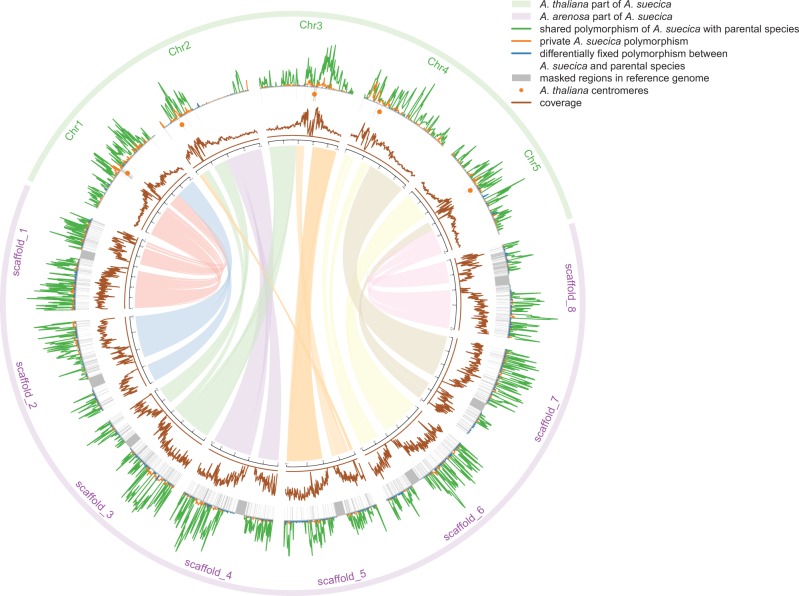
Polymorphism density (outer graph) and sequencing coverage (inner, brown graph) along the chromosomes of *A. suecica* (shown on the outer rim, with the five *A. thaliana* chromosomes indicated in green and the eight *A. lyrata* reference genome scaffolds indicated in purple). The polymorphism density (number of SNPs per aligned site) along the genome is shown separately for shared (green), private (orange), and differentially fixed polymorphism (blue). A large non-polymorphic region is located between 7.8 and 16.2 Mbp on chromosome 2 of the *A. thaliana* portion of *A. suecica*. Links between the *A. thaliana* and *A. lyrata* reference genomes (center) are adapted from (Hu et al. 2011).

Previous results have suggested that *A. suecica* had a unique origin, undergoing an extreme bottleneck that completely wiped out ancestral polymorphism, at least in the *A. thaliana* portion of the genome (Jakobsson et al. 2006). We were thus very surprised to find that 89% of identified polymorphisms for the *A. thaliana* portion of *A. suecica* are shared with contemporary *A. thaliana*. A similar result was obtained for the *A. arenosa* portion of the genome: 91% of polymorphic sites are shared with *A. arenosa* (fig. 1, supplementary fig. S2, Supplementary Material online). This amount of shared or, rather, retained ancestral variation clearly contradicts the previously suggested unique origin of *A. suecica* (Jakobsson et al. 2006), especially since *A. thaliana* was already selfing when it contributed to *A. suecica* (see below, supplementary fig. S3, Supplementary Material online), and thus is unlikely to have contributed more than one allele at each locus. Most A. thaliana individuals are almost completely homozygous, and although outcrossing occurs, even a single generation of selfing renders half the genome homozygous (furthermore, as we shall see below, many regions of the genome harbor more than two ancestral haplotypes and must therefore have more than a single ancestor).

Nevertheless, there are clear traces of a major bottleneck, presumably associated with the origin of the new polyploid species from a relatively small number of founders. Not only is the overall level of polymorphism strongly reduced (to roughly 30 and 12% of that of *A. thaliana* and *A. arenosa*, respectively), but also non-synonymous and putatively deleterious alleles are present at higher frequencies than in the parental species (supplementary fig. S4*A* and *B*, Supplementary Material online)—as expected as a consequence of drift during a bottleneck. We note, however, that purifying selection appears to have been operating after the establishment of the species: among polymorphisms private to *A. suecica* (i.e., polymorphisms that must have arisen in the species), non-synonymous ones are again biased toward rare alleles (supplementary fig. S4*C* and *D*, Supplementary Material online).

There are also large chromosomal regions almost devoid of variation (fig. 1) in the *A. suecica* genome. While some of these may reflect selective sweeps in the new species, a simpler explanation is that they are a consequence of the foundation bottleneck. Indeed, the ubiquity and size of these regions will make it very difficult to find any genuine selective sweeps. The largest region, on the second chromosome of the *A. thaliana* portion of the genome, covers most of the long arm (∼8 Mb). We can use this to estimate how old *A. suecica* is. Under the assumption that the small amount of polymorphism that does exist in this region has been generated solely by new (i.e., non-ancestral) mutations (only 4.5% of polymorphism in this genomic region is shared with *A. thaliana*, which should be compared with the genome-wide average of 89%, see above), we estimate that the bottleneck occurred ∼16 Kya (95% CI [14.1–18.4 Kya]: other bottlenecked regions give similar results; see “Materials and Methods” section). Consistent with this, estimates of the effective population size over time (using MSMC; Schiffels and Durbin 2014) based on the full data point to a sharp decline following the last glacial maximum roughly 22 Kya (supplementary fig. S5, Supplementary Material online). The decline is particularly noticeable in the *A. arenosa* portion of the genome, which is expected given that the ancestral species was an obligate outcrosser, implying that this portion underwent a transition to selfing as well.

**Fig. 2:**
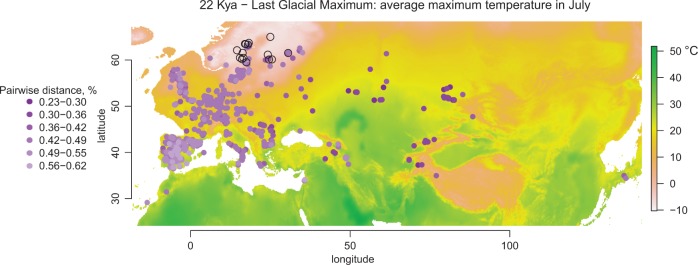
The *A. thaliana* accessions most closely related to *A. suecica* are found in northern and central Eurasia (indicated by the dark violet circles). The background color of the map indicates the average maximum temperature in July during the last glacial maximum (Hijmans et al. 2005). The present distribution of *A. suecica* (Fennoscandinavia; *A. suecica* sampling locations are indicated with open black circles) was covered by ice during this time, experiencing temperatures below 0 °C in July.

The next question is where *A. suecica* originated. We sought the ancestral *A. thaliana* population in the worldwide collection of sequenced *A. thaliana* genomes (TG Consortium 2016). Based on pairwise sequence divergence, the most closely related *A. thaliana* accessions appear to be found around the Ural Mountains, and in northern and central Eurasia (fig. 2). Clustering accessions using ADMIXTURE (Alexander et al. 2009), similarly groups *A. thaliana* accessions from northern and central Eurasia with *A. suecica*, suggesting a shared past (supplementary fig. S6, Supplementary Material online). Thus *A. suecica* is not most closely related to the *A. thaliana* with which it currently coexists, and the ancestral population must have been elsewhere. Indeed, because the Fennoscandinavian region was covered by ice until ∼6 Kya (fig. 2; http://www.worldclim.org/; last accessed January 2017; Hijmans et al. 2005), it is obvious that both species must be recent immigrants. Given that Swedish *A. thaliana* (unlike Swedish *A. suecica*) do not appear to be particularly closely related to Russian *A. thaliana* (1001 Genomes Consortium 2016), a plausible scenario is that *A. thaliana* mainly reached Scandinavia from the south, via present-day Denmark (1001 Genomes Consortium 2016), while *A. suecica* took the northern route, via present-day Finland.

**Fig. 3:**
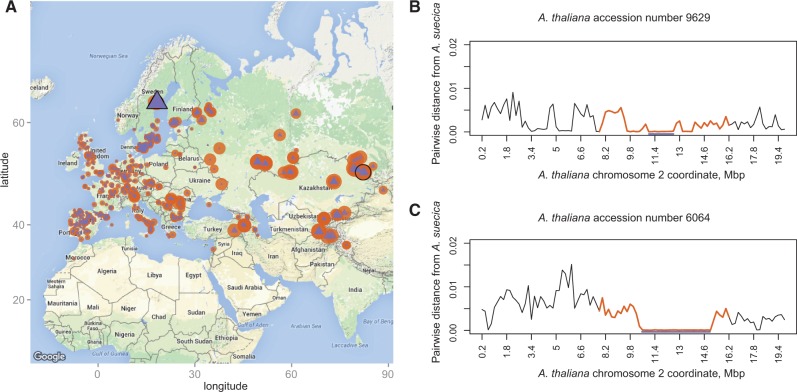
The relationship between *A. suecica* and *A. thaliana* accessions in the bottlenecked region corresponding to *A. thaliana* chromosome 2 (fig. 1). (***A***) The size of the orange circles is inversely proportional to the average pairwise distance between *A. thaliana* and *A. suecica* w.r.t. the bottlenecked region of interest; this distribution is similar to the genome-wide pattern shown in figure 2. The closest accession (9,629) is highlighted with a black circle. The size of the violet triangles is proportional to the length of a haplotype that is shared with *A. suecica*. The accession with the longest haplotype (6,064) is highlighted with a black triangle. (***B***,***C***) The average pairwise distance between 9,629 and 6,064, respectively, and *A. suecica*. The violet line shows the position of the longest haplotype that is shared with *A. suecica*; the orange lines show the region inherited from one founder in *A. suecica* and used to calculate the pairwise distance on **A**.

Interestingly, although *A. suecica* is clearly most closely related to Russian *A. thaliana* (fig. 2), the largest shared haplotype was found in *A. thaliana* from northern Sweden. An almost ∼4 Mb segment of the ∼8 Mb bottlenecked region on chromosome 2 (fig. 1) appears to be shared (i.e., identical-by-descent) with an *A. thaliana* accession from northern Sweden (fig. 3). However, this accession is not particularly closely related to *A. suecica* in any other sense, and the bottlenecked region does not show a pattern of relatedness different from the genome-wide pattern (fig. 3*A*–*C*). Since recent admixture is extremely unlikely (*A. thaliana* and *A. suecica* do not produce fertile offspring), one explanation is that this is a case of ancestral haplotype sharing, with the extreme length in northern Sweden being another facet of the generally much more extensive haplotype sharing and linkage disequilibrium in *A. thaliana* in this part of the world (Long et al. 2013). Simply put, there has effectively been less recombination in northern Swedish *A. thaliana* since the last glaciation, and this has led to greater haplotype sharing with ancient *A. thaliana*, and hence with *A. suecica*. Consistent with this interpretation, there is extensive haplotype sharing in this chromosomal region throughout Europe (fig. 3*A*).

Alternatively, the extensive haplotype sharing in northern accessions is a consequence of some *A. thaliana* having migrated to Sweden together with *A. suecica* via a northern route, and since admixed with *A. thaliana* coming from the south. Interestingly, the *A. thaliana* accession closest to *A. suecica* (by average pairwise divergence) is from the Altai mountain range in Central Asia (fig. 3*B*), a region which served as a refugium for many species (including humans; Reich et al. 2010; Prufer et al. 2014) during the glacial interchanges (Tarasov et al. 2000; Pavelkova Ricankova et al. 2014). One can thus imagine a scenario wherein a local *A. thaliana* population, which contributed to the formation of *A. suecica*, occupied new territories following the retraction of the ice sheets alongside its hybrid progeny. Where this would have taken place is far from clear. While the closest relatives on the *A. thaliana* side are currently found in Central Asia, there is no evidence for the other parent of *A. suecica*—*A. arenosa*—in this region. Thus, the most likely scenario may be that *A. suecica* originated somewhere in Eastern Europe and migrated to Fennoscandinavia following the retracting ice, while its parental *A. thaliana* population additionally spread into Central Asia.

**Fig. 4:**
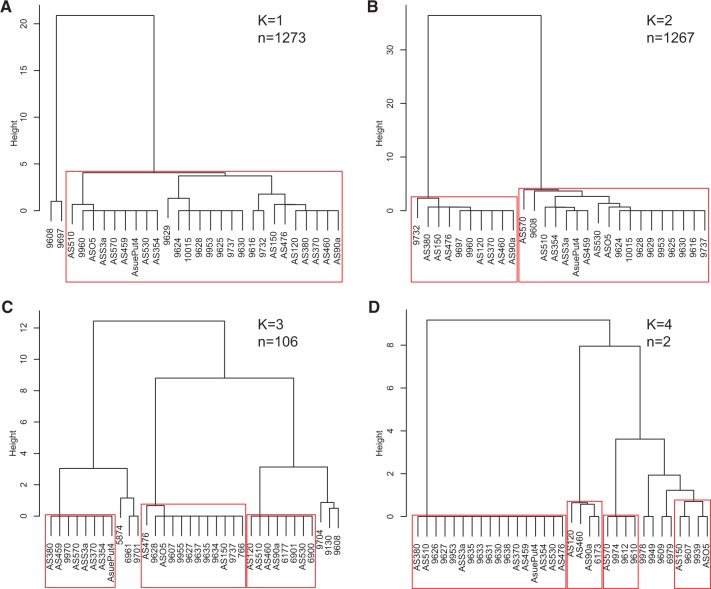
Number of founder haplotypes in *A. suecica*. (***A–D***) Examples of the different number of founder haplotypes in *A. suecica*. *A. suecica* accessions are divided into clusters (red boxes) which also include accessions of *A. thaliana* and are strongly supported by *P*-values following multiscale bootstrap resampling (“Materials and Methods” section).

Next, we considered the number of *A. thaliana* individuals that contributed to the founding of *A. suecica*. As we have seen, the number of founding haplotypes is one for several regions of the genome, most noticeably on chromosome 2 (figs. 1 and3). As it happens, another example (as in fig. 4*A*) is a region located at the top of chromosome 4, which harbors the four loci previously used to conclude that *A. suecica* likely had a unique origin (Jakobsson et al. 2006). This conclusion was thus correct for this region, but is clearly incorrect for most of the genome. To gain further insight into the number of founders, we searched the genome for ancestral haplotype blocks shared between *A. suecica* and *A. thaliana* (using PLINK; see “Materials and Methods” section). We identified 1,273 haplotype blocks for which all *A. suecica* accessions fell into a single cluster of almost identical haplotypes, which we interpret as sharing a single founder haplotype (fig. 4*A*). Similarly, we found 1,267 blocks for which the accessions can be clearly divided into 2 haplotype clusters; 106 for which they can be divided into 3 haplotype clusters, and 2 for which there were 4 clusters (fig. 4*B–D*). As a consistency check, we estimated the divergence time for haplotypes belonging to the same founder haplotype and obtained a very similar estimate to that reported above for the chromosome 2 region (95% CI [15.1–16.6 Kya], see “Materials and Methods” section).

The observed numbers of founder haplotypes do not directly correspond to the number of *A. thaliana* founders, as lineages may have been lost through drift (Nordborg 1998). Of course the number of haplotypes provides a lower bound, and we can therefore conclude that at least four founding individuals contributed (under the assumption that the founders were inbred, which is likely). To see if it is possible to be more precise, we simulated gene genealogies under linear growth models with varying numbers of founding individuals at the estimated time of origin (∼16 Kya). These simulations showed that the observed distribution of founder lineages is compatible with a wide range of parameters (supplementary fig. S7, Supplementary Material online), and it is therefore unlikely that we will be able to refine our estimate of the number of founders further.

Finally, we considered the transition to selfing in *A. suecica*. *A. suecica* is self-compatible (Säll et al. 2004), which agrees with the general association between polyploidy and selfing in plants (Barringer 2007). Selfing often evolves through the loss of the self-incompatibility system, which is controlled by the S-locus in flowering plants (Barrett 2002). In the genus *Arabidopsis*, the tightly linked male *SCR* (S-locus cysteine-rich protein—present in the pollen coat) and female *SRK* (S-locus receptor kinase—expressed on the surface of the stigma) determine the specificities of the self-recognition system: the male gene coding for the ligand and the female gene coding for the receptor (Takayama and Isogai 2005). Recognition of *SCR* by the *SRK* protein triggers a downstream signaling pathway that prevents pollen tube growth (Comai et al. 2000; Takayama and Isogai 2005; Chapman and Goring 2010). In the predominantly selfing *A. thaliana*, the S-locus is nonfunctional due to several loss-of-function mutations (Nasrallah et al. 2002; Liu et al. 2007; Sherman-Broyles et al. 2007; Tang et al. 2007; Shimizu et al. 2008; Boggs et al. 2009; Tsuchimatsu et al. 2010).

“Synthetic *A. suecica*” F1 hybrids, produced by fertilizing colchicine-induced tetraploids of *A. thaliana* with pollen from naturally tetraploid *A. arenosa*, were not immediately selfing, and exhibited many abnormal phenotypes compared to natural *A. suecica* (Chen et al. 1998; Comai et al. 2000), however, they became increasing self-compatible after several rounds of forced self-pollination (Z.J. Chen, personal communication). However, a single *A. arenosa* collect (*Care-1*) was used in these crosses, and it is thus not known if other combinations of parents produce fully self-compatible hybrids.

In our sample of natural *A. suecica*, we found that the S-locus inherited from *A. thaliana* is fixed for the 213-bp inversion in the *SCR* gene (supplementary fig. S3, Supplementary Material online) that is suggested to have led to loss of self-incompatibility in *A. thaliana* (Tsuchimatsu et al. 2010). Therefore, *A. thaliana* was almost certainly already self-compatible when it contributed to *A. suecica*, supporting the notion that the transition to selfing is more ancient (Bechsgaard et al. 2006; Tang et al. 2007; Hu et al. 2011).

**Fig. 5:**
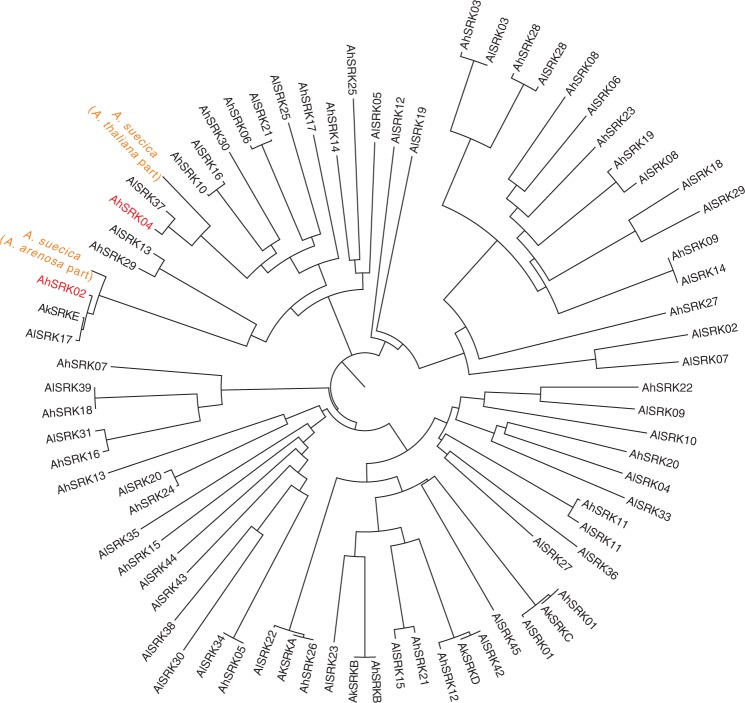
The phylogeny of the *SRK* sequences belonging to *A. lyrata* (AlSRK), *A. halleri* (AhSRK), *A. kamchatica* (AkSRK), and *A. suecica*. The alignment is adapted from Tsuchimatsu et al. (2012) and the phylogeny was generated using a neighbor-joining algorithm.

*A* *rabidopsis* *arenosa* is an obligate outcrosser (Säll et al. 2004), hence *A. suecica* should have inherited fully functional S-alleles from *A. arenosa*, and these must somehow have been rendered nonfunctional or silenced. *Arabidopsis* S-alleles have a complex dominance hierarchy, determined by a small RNA regulatory network (Tarutani et al. 2010; Durand et al. 2014) in which small RNAs from some S-alleles can silence expression of *SCR* on other S-alleles. The S-locus is a classic example of a long-term balancing selection, with suppressed recombination between SRK and SCR leading to highly diverged S-allele shared across species (Mable et al. 2003, 2004; Castric and Vekemans 2004; Castric et al. 2008; Llaurens et al. 2008). In other words, an S-allele from *A. arenosa* may be more closely related to, for example, an S-alleles from *A. halleri*, than to other *A. arenosa* alleles. The *A. thaliana* S-allele found in *A. suecica* is predicted (based on cross-species alignments; see fig. 5 and “Materials and Methods” section) to be an ortholog of the *A. halleri* S-haplogroup 4, whereas the *A. arenosa* S-allele found in *A. suecica* is orthologous to S-haplogroup 2. Based on crosses in *A. halleri*, the former has been shown to be dominant over the latter (Llaurens et al. 2008), meaning that in *A. suecica* the S-allele at the locus inherited from *A. arenosa* is predicted to be transcriptionally silenced by the S-allele at the locus inherited from *A. thaliana*. Further investigation showed that the main players ensuring such a dominance mechanism (miRNA-producing loci and target sites) are most probably functional in *A. suecica* (“Materials and Methods” section, supplementary fig. S8, Supplementary Material online).

Moreover, mapping *A. suecica* to a complete BAC-sequence of *A. halleri* S-allele 2 (“Materials and Methods” section) revealed that the S-locus inherited from *A. arenosa* is fixed for a single S-allele, which exhibited a frame-shift mutation in *SCR* that is predicted to lead to loss of function (supplementary fig. S9, Supplementary Material online). It is therefore possible that *A. suecica* was immediately at least partly self-compatible due to the non-functional allele from *A. thaliana* being dominant over the functional allele from *A. arenosa*, and that the loss-of-function mutation was fixed later—it could either have been fixed by drift, or by selection. Independent of explanation, this mutation rendered the species fully self-compatible. In support of the former explanation, however, there is no sign of a selective sweep in the S-allele region from *A. arenosa* (supplementary fig. S10, Supplementary Material online).

In conclusion, whole-genome sequencing of 15 natural *A. suecica* accessions from the entire species range revealed that the species was almost certainly founded by multiple hybridizations between *A. thaliana* (as the mother) and tetraploid *A. arenosa* (as the father), which is in line with generalizations made for the origins of the other well-established allotetraploid species from multiple founders (Soltis et al. 2004; Shimizu-Inatsugi et al. 2009; Vallejo-Marin et al. 2015). Any scenario involving a single origin would have to invoke a completely outcrossed parent on the *A. thaliana* side (to explain genome-wide allele sharing), and subsequent gene flow between species with dramatically different karyotypes (to explain the regions of the genome that harbor more than two ancestral haplotypes). The species appears to have originated somewhere in central Eurasia in conjunction with the last glacial maximum, and subsequently migrated to Fennoscandinavia. A possibility is that hybridization was facilitated by the production of an unusually high frequency of unreduced *A. thaliana* ovules in a population containing both parental species, perhaps as a result of environmental stress (Madlung 2013; Vanneste, Maere, et al. 2014; Mason and Pires 2015; Zhou et al. 2015). The transition to self-fertilization may have been facilitated by the dominance of the non-functional S-allele from *A. thaliana* over the S-allele from *A. arenosa*, followed by inactivation of the latter.

## Materials and Methods

### DNA Extraction, Library Preparation, and Sequencing

Whole genomic DNA was extracted from fresh leaf material using DNEasy Plant Mini Kit (Qiagen). Total genomic DNA libraries were prepared using a slightly modified Illumina Genomic DNA Sample Prep protocol. In brief, 100–200 ng DNA was fragmented by sonication with Bioruptor (Diagenode); the peak of fragment sizes was ∼400 bp. End-repair of sheared DNA fragments, A-tailing and adapter ligation were done with the NEXTflex DNA Sequencing Kit (Bio Scientific). Adaptor-modified DNA was resolved on 1.5% low melt agarose (Peqlab) gel. For size selection of the library, DNA was excised from the gel with the size range from 300 to 500 bp. The paired-end DNA libraries were amplified for 10–12 cycles by PCR with VeraSeq PCR Mix (Biozym). Libraries were sequenced in 100-bp paired-end mode on Illumina HiSeq 2000 Analyzers using manufacturer’s standard cluster generation and sequencing protocols.

Raw sequencing data for 12 new *A. suecica* accessions were uploaded to NCBI SRA under BioProject ID PRJNA309929; data for 3 additional accessions were already published (Novikova et al. 2016) and are available under BioProject ID PRJNA284572 (supplementary table S1, Supplementary Material online).

### Read Mapping and Variants Discovery

We mapped *A. suecica* reads to the *A. thaliana* (TAIR10) and the *A. lyrata* (v1.0) references simultaneously using the BWA-MEM algorithm from BWA (Li and Durbin 2009) (version 0.7.8) with an increased penalty of 15 for unpaired read pairs. The *A. lyrata* reference genome is the closest available reference to *A. arenosa*, and the successful mapping of *A. suecica* to a combination of the *A. thaliana* and *A. lyrata* genomes has been reported before (Henry et al. 2014). We used Samtools (Li et al. 2009) (version 0.1.19) to sort, index and remove potential duplications from the PCR amplification step of library preparation. We then performed a local realignment with IndelRealigner from Genome Analysis Toolkit (McKenna et al. 2010; DePristo et al. 2011) (version 3.3.0). After filtering for uniquely aligned reads with Samtools, we called sites and variants using GATK UnifiedGenotyper with default parameters. We combined called sites from all *A. suecica* samples with CombineVariants from GATK and annotated called sites using SNPeff (Cingolani et al. 2012). In order to decrease the number of variant calls from any misaligned homoeologous regions, we filtered out all the heterozygous calls, which were present in the population sample more than once.

We used previously published raw data from *A. arenosa* clade samples (Novikova et al. 2016). *A. arenosa* reads were mapped to the *A. lyrata* (v1.0) reference, using the pipeline described above. All confident *A. arenosa* calls, including variants, were combined with the *A. suecica* calls for the *A. lyrata* component of the reference used for the mapping of *A. suecica*. The *A. thaliana*-derived portion of *A. suecica* was compared throughout the study with variant calls of Eurasian accessions taken from the 1001 *Arabidopsis* genome project (1001 Genomes Consortium 2016).

### Ancestral *A. thaliana* Population(s) for *A. suecica*

Shared, private, and differentially fixed polymorphism in *A. suecica* were compared with the parental species (fig. 1) and were calculated for all sites, for which data were available for at least 80% of individuals in both populations for each comparison. Polymorphism density in figure 1 represents the number of polymorphic sites per aligned site in 200-kb windows along the genome. The R library ‘circlize’ was used to visualize data along the chromosomes of *A. suecica*.

Pairwise divergence between *A. thaliana* and *A. suecica* accessions was calculated with custom scripts as a percentage of diverged sites from all aligned sites, excluding indels. Figure 2 shows minimal divergence between each *A. thaliana* accession and all *A. suecica* accessions. Maximum likelihood estimates of individual ancestries (with *K* = 9 for the number of clusters) were done via ADMIXTURE (Alexander et al. 2009), allowing for missing data. The R library ‘ggmap’ was used to visualize the data points on Google Maps. The R library ‘raster’ was used for visualizing the climate data from http://www.worldclim.org/ with a resolution of 10 min.

In figure 3*B* and *C*, the average pairwise distance between 9,629 and 6,064 was calculated in 200-kb windows along the genome. The violet lines in figure 3*B* and *C* show the position of the longest haplotype that is shared with *A. suecica* and is defined by using a threshold of a 0.1% error rate along the region of interest (indicated in orange).

### Number of Founder Haplotypes in *A. suecica*

We divided the *A. thaliana* portion of the *A. suecica* genome into 200-kb windows, and estimated pairwise divergence between all *A. thaliana* and *A. suecica* accessions. We choose the five closest *A. thaliana* accessions for each *A. suecica* accession, which resulted in a range of 5–43 unique *A. thaliana* accessions, depending on the examined interval. For each interval, we calculated haplotype blocks and all possible haplotype phases for *A. suecica* together with the selected *A. thaliana* accessions, using PLINK (Purcell et al. 2007) with default parameters. Using consensus sequences of the most likely haplotype phases for each individual, we performed hierarchical clustering and calculated an uncertainty level for each cluster with the R package ‘pvclust’ (Suzuki and Shimodaira 2006) (method.hclust=“ward.D2”). The uncertainty of each cluster was assessed using an ‘AU’ (Approximately Unbiased) *P*-value, which is computed by multiscale bootstrap resampling. In order to estimate the number of founder haplotypes in *A. suecica*, we counted, for each haplotype, the number of clusters with an AU greater than 99%, where *A. suecica* and *A. thaliana* accessions are present in the same cluster.

We ran coalescent simulations using the R library ‘scrm’ (analogous to ms; Hudson 2002) under a linear growth model with a varying θ (measured in 4N_0_ generations) and population size at time *t*: *N_t_*_ _=_ _n*N_0_, where *N*_0_ is the contemporary population size of *A. suecica* and *N_t_* is the number of founders for *A. suecica*. These parameters were uniformly distributed (*N*_0_ varied from 1,000 to 100,000) and (*N_t_* varied from 1 to 1,000 for each *N*_0_) and result in a total of 100,000 MS runs. For each MS run, 2,648 gene genealogies were generated, where we calculated the number of ancestral lineages for time *t* = 16 Kya, which match our estimate for the origin time of *A. suecica* (supplementary fig. S7, Supplementary Material online). A comparison with the observed number of ancestral lineages for 2,648 loci (fig. 4) was conducted via a least squared distance method. An additional 1,000,000 simulations were run with a fixed *N*_0_ of 5,000 and from this we chose 1,000 simulations that were close to the observed data, allowing a posterior distribution of the *N_t_* parameter to be inferred.

### Dating the Origin of *A.suecica*

Our estimation of the time of origin of *A. suecica* was based on the assumption that at loci inherited from one founder, the population diversity within *A. suecica* is generated solely by new mutations. Therefore, we can estimate the origin time of *A. suecica* simply as *π*/2 *μ*, where *π* is nucleotide diversity within *A. suecica* at the single founder region, *μ* is mutation rate and the generation time is 1 year (*A. suecica* is an annual plant). Here, we take the mutation rate of *A. suecica* to equal that of *A. thaliana*: 7 × 10^−^^9^ base substitutions per site per generation (Ossowski et al. 2010). We calculated the origin time of *A. suecica* as the expected coalescence time within accessions at loci which belong to the same founder haplotype: 95% CI [15.1–16.6 Kya]. Confidence intervals for the median of the distribution were calculated using the basic bootstrap method in the R package ‘boot’. We obtained a similar result for the estimated origin time of *A. suecica* (95% CI [14.1–18.4 Kya]) by applying the same logic at the largest single founder region on the second chromosome of the *A. thaliana* portion of the *A. suecica* genome (between 7.8 and 16.2 Mbp). Nucleotide diversity was calculated in 200-kb windows along this region.

Coalescent rates and the scaled population size over time were inferred using MSMC (Schiffels and Durbin 2014) for six combinations of four randomly chosen *A. suecica* accessions separately for mapping to *A. thaliana* and *A. lyrata* (representing the *A. arenosa* portion of the *A. suecica* genome) chromosomes of the combined reference genome. Only intervals with a continuous coverage over 10 kb were chosen for the analysis.

### Mechanism of Self-Compatibility in *A. suecica*

Combining *de novo* assembly and alignment tactics, we assigned S-haplogroups to *A. thaliana-* and *A. arenosa*-derived S-alleles in *A. suecica* using partial *SRK* sequences (see below). *A. thaliana*-derived and *A. arenosa*-derived S-alleles of *A. suecica* appear to be orthologous to corresponding *A. halleri* S-alleles 4 (AhS04) and 2 (AhS02) (fig. 5).

AhS02 and AhS04 are members of the second most recessive class of *Arabidopsis* S-haplogroups (Durand et al. 2014). However, the inferred pollen phenotype of *A. halleri* plants with a heterozygous AhS02/AhS04 genotype was that of AhS04 (Llaurens et al. 2008). This suggests that the *A. thaliana*-derived S-haplogroup (an ortholog of AhS04) could be partially dominant over the *A. arenosa*-derived S-haplogroup (an ortholog of AhS02) in *A. suecica*; and that the *A. suecica* pollen phenotype should correspond to the *A. thaliana*-derived *SCR*, which is truncated and allows for selfing. Such a combination of S-haplogroups also appears to be present in all the analyzed *A. suecica* accessions and, most probably, fixed in the *A. suecica* species.

Mapping *A. suecica* to a complete BAC-sequence of *A. halleri* S-allele 2 (see below) revealed that a loss-of-function mutation at the *SCR* gene (supplementary fig. S9, Supplementary Material online) is fixed in *A. suecica*, while functionally and structurally important residues (supplementary fig. S11, Supplementary Material online) are conserved in the *SRK* gene. It is therefore possible that *A. suecica* became a selfer following the frame-shift mutation in *SCR*. However, it is also possible that silencing of the *A. arenosa*-derived S-allele by the *A. thaliana*-derived S-allele provided an immediate selfing opportunity for *A. suecica*, followed by the subsequent pseudogenization of the *SCR* gene, making *A. suecica* an irrevocable selfer. In line with this hypothesis, we found that *mir867* expressed in the *A. thaliana* haplotype A of Col-0 as well as the corresponding *A. halleri* haplotype 4, which is predicted to be able to target the *A. halleri* haplotype 2 is fully identical to the mature miRNA-producing portion in *A. suecica* (supplementary fig. S8, Supplementary Material online). Its target sequence in the orthologous *A. suecica SCR02* is also fully identical, suggesting that the silencing mechanism could have been active at the speciation time of *A. suecica*. Target sites of sRNA reads produced by *mir867* of AhS04 and the *A. thaliana* haplotype A were predicted by mapping to the AhSCR02 genomic sequence using a modified Smith-Waterman algorithm and a threshold of 18 (Durand et al. 2014). sRNA sequencing data were from (Durand et al. 2014) for Ah04 in *A. halleri* (GSM1378105) and from (Montgomery et al. 2008) (ago1-25, GSE13605), (Mi et al. 2008) (AGO4-IP, GSE10036), and (Zheng et al. 2010) (rdr6, GSE23439) for haplotype A in *A. thaliana*.

### Assembly of the *A.suecica* Accession ASS3a and the Assignment of S-Alleles

We assembled one *A. suecica* accession (ASS3a) that possessed the highest number of Illumina reads, using SOAPdenovo2 (Luo et al. 2012) (127mer version 1.4.10) with a kmer length equal to 73. We used all the reads, both at the contig and scaffold assembly level (asm_flags = 3). The resulting assembly had an N50 length of scaffolds equal to 38,763 bp.

Using the *SCR* sequence from *A. thaliana* (Col-0) as a query (Shimizu et al. 2008), we searched in our *de novo A. suecica* assembly for the scaffold containing *SCR*-like sequences using Blast (v. 2.2.28) and applying penalties for the opening and extension of gaps equal to 2 and 1, respectively. With this, we identified scaffold3258 as the scaffold that contains the *A. suecica SCR* sequence for the *A. thaliana*-derived portion of the genome.

We obtained available *SRK* sequences for *A. thaliana*, *A. halleri*, and *A. lyrata* (Schierup et al. 2001; Charlesworth et al. 2003; Bechsgaard et al. 2006; Castric and Vekemans 2007; Tang et al. 2007; Castric et al. 2008, 2010; Tsuchimatsu et al. 2012). Using those *SRK* sequences as a query, we searched for the scaffolds containing *SRK*-like sequences in our *A. suecica* assembly using Blast with the same parameters. Combining the percent of identity with the bit score, we identified C3267705 and scaffold11240 as scaffolds containing the *SRK* sequences for the *A. thaliana* and *A. arenosa* portions of *A. suecica*, respectively. In order to assign the S-haplogroups in the ASS3a *A. suecica* accession, we incorporated the obtained *SRK* sequences from C3267705 and scaffold11240 scaffolds into the *SRK* alignment (Tsuchimatsu et al. 2012). We used CLC Main Workbench v7.0.2 (CLC bio, Aarhus, Denmark) to align the *SRK* sequences and applied default parameters for a ‘slow’ alignment: with gap open and extension cost being 10.0 and 1.0, respectively. A neighbor-joining tree from the *SRK* alignment was constructed using the same software, applying the Jukes-Cantor nucleotide distance as a measure.

In order to check whether all *A. suecica* accessions carry the same S-haplogroups, we included the partially assembled *A. arenosa* S-locus sequence from our *A. suecica* assembly (scaffold11240) to the combined reference genome of *A. thaliana* (TAIR10) and *A. lyrata* (v1.0) and mapped all the *A. suecica* accessions to this novel reference. Mapping was conducted using the pipeline described above, however, we did not filter for ‘primary’ aligned reads. The same pipeline was used for mapping of *A. suecica* accessions to the BAC-sequence of *A. halleri* S-allele 2, together with the *A. thaliana* and *A. lyrata* reference genomes. Consensus sequences of *SCR* and *SRK* genes were obtained with GATK FastaAlternateReferenceMaker (McKenna et al. 2010; DePristo et al. 2011), aligned with MAFFT (Katoh and Standley 2013) (version 7) and visualized with JalView (Waterhouse et al. 2009).

### Construction of BAC Libraries

High Molecular Weight (HMW) DNA was prepared from young leaves of *Arabidopsis halleri* var. P21M53. For the extraction, 20 g of frozen leaf tissue was grounded to a powder in liquid nitrogen with a mortar and pestle in order to prepare megabase-size DNA embedded in agarose plugs. HMW DNA was prepared as described by Peterson et al. (2000) and modified as described by Gonthier et al. (2010). Embedded HMW DNA was partially digested with *Hind*III (New England Biolabs, Ipswich, MA), and subjected to two size selection steps by pulsed-field electrophoresis, using a BioRad CHEF Mapper system (Bio-Rad Laboratories, Hercules, CA), and ligated to pIndigoBAC-5 *Hind*III-Cloning Ready vector (EpicentreBiotecnologies, Madison, WI). Pulsed-field migration programs, electrophoresis buffer, and ligation desalting conditions were performed according to Chalhoub et al. (2004). The BAC library is composed by 18,432 clones with a mean insert size of 110 kb and represents 6 genome equivalents.

### PacBio RS II Sequencing and Assembly of the S-Locus

2 µg of BAC clone Aha_P21M53_40F08 were pooled with 11 other BAC clones DNA to obtain a total amount of 24 µg. One library was generated using the standard Pacific Biosciences library preparation protocol for 8–12 kb libraries. This library was sequenced in one PacBio RS II SMRT Cell using the P4 polymerase in combination with the C2 chemistry (sequencing service following the standard operating procedures was provided by IGM Genomic Center).

Assembly of the PacBio RS II reads was performed following the HGAP workflow. The SMRT Analysis (v2.2.0) software suite was used for HGAP implementation (https://github.com/PacificBiosciences/Bioinformatics-Training/wiki/HGAP; last accessed January 2017). Reads were first aligned using BLASR (“Blasr on Pacific Biosciences repository website,” n.d.; Chaisson and Tesler 2012) against “*Escherichia coli* str. K12 substr. DH10B, complete genome”. Identified *E. coli* reads and low quality reads (read quality <0.80 and read length <500 bp) were removed from data used for the BAC clone sequences assembly. Vector sequences were trimmed as part of the assembly process. Each BAC assembly was individualized by matching its BES to the ends of assembled sequences using BLAST. Annotation of the *SRK* and *SCR* genes followed (Goubet et al. 2012) and annotation of small RNA precursors followed (Durand et al. 2014).

## Supplementary Material

Supplementary data are available at *Molecular Biology and Evolution* online.

## Supplementary Material

Supplementary Data
