# Supplementary material for: Genome Sequencing Reveals the Origin of the Allotetraploid Arabidopsis suecica
Source: Mol Biol Evol. 2017 Jan 12;34(4):957–68. doi: 10.1093/molbev/msw299 (PMC5400380; doi:10.1093/molbev/msw299)
Supplement: Supplementary Data [file msw299_Supp.docx]

**Supplementary Figures and Tables**

**Supplementary table S1.** Analyzed *A. suecica* accessions.

| **accession** | **lat(N)** | **long(E)** | **Country** | **# reads** | **mapped reads after filtering, %** | **BioProject ID** | **BioSample_accession** |
| --- | --- | --- | --- | --- | --- | --- | --- |
| AS120 | 61.54 | 16.33 | Sweden | 97,785,320 | 77.24 | PRJNA309929 | SAMN04442133 |
| AS150 | 62.10 | 14.56 | Sweden | 176,097,226 | 78.40 | PRJNA309929 | SAMN04442134 |
| AS354 | 63.43 | 17.27 | Sweden | 138,867,478 | 77.58 | PRJNA309929 | SAMN04442135 |
| AS370 | 64.95 | 25.23 | Finland | 125,410,546 | 78.23 | PRJNA309929 | SAMN04442136 |
| AS380 | 60.24 | 25.12 | Finland | 215,698,776 | 72.57 | PRJNA309929 | SAMN04442137 |
| AS459 | 60.19 | 16.12 | Sweden | 316,754,654 | 78.55 | PRJNA284572 | SRS977003 |
| AS460 | 60.48 | 15.48 | Sweden | 41,733,088 | 76.66 | PRJNA309929 | SAMN04442138 |
| AS476 | 59.51 | 17.49 | Sweden | 90,375,686 | 79.24 | PRJNA309929 | SAMN04442139 |
| AS510 | 60.11 | 24.59 | Finland | 76,672,068 | 77.08 | PRJNA309929 | SAMN04442140 |
| AS530 | 61.15 | 24.20 | Finland | 47,870,552 | 76.90 | PRJNA309929 | SAMN04442141 |
| AS570 | 60.46 | 16.57 | Sweden | 41,636,570 | 77.31 | PRJNA309929 | SAMN04442142 |
| AS90a | 63.47 | 17.05 | Sweden | 256,747,096 | 77.23 | PRJNA309929 | SAMN04442143 |
| ASO5 | 63.70 | 18.25 | Sweden | 369,320,826 | 78.35 | PRJNA284572 | SRS977001 |
| ASS3a | 63.33 | 17.98 | Sweden | 418,718,928 | 73.58 | PRJNA284572 | SRS977000 |
| AsuePut4 | 61.51 | 30.58 | Russia | 87,821,588 | 78.03 | PRJNA309929 | SAMN04442144 |


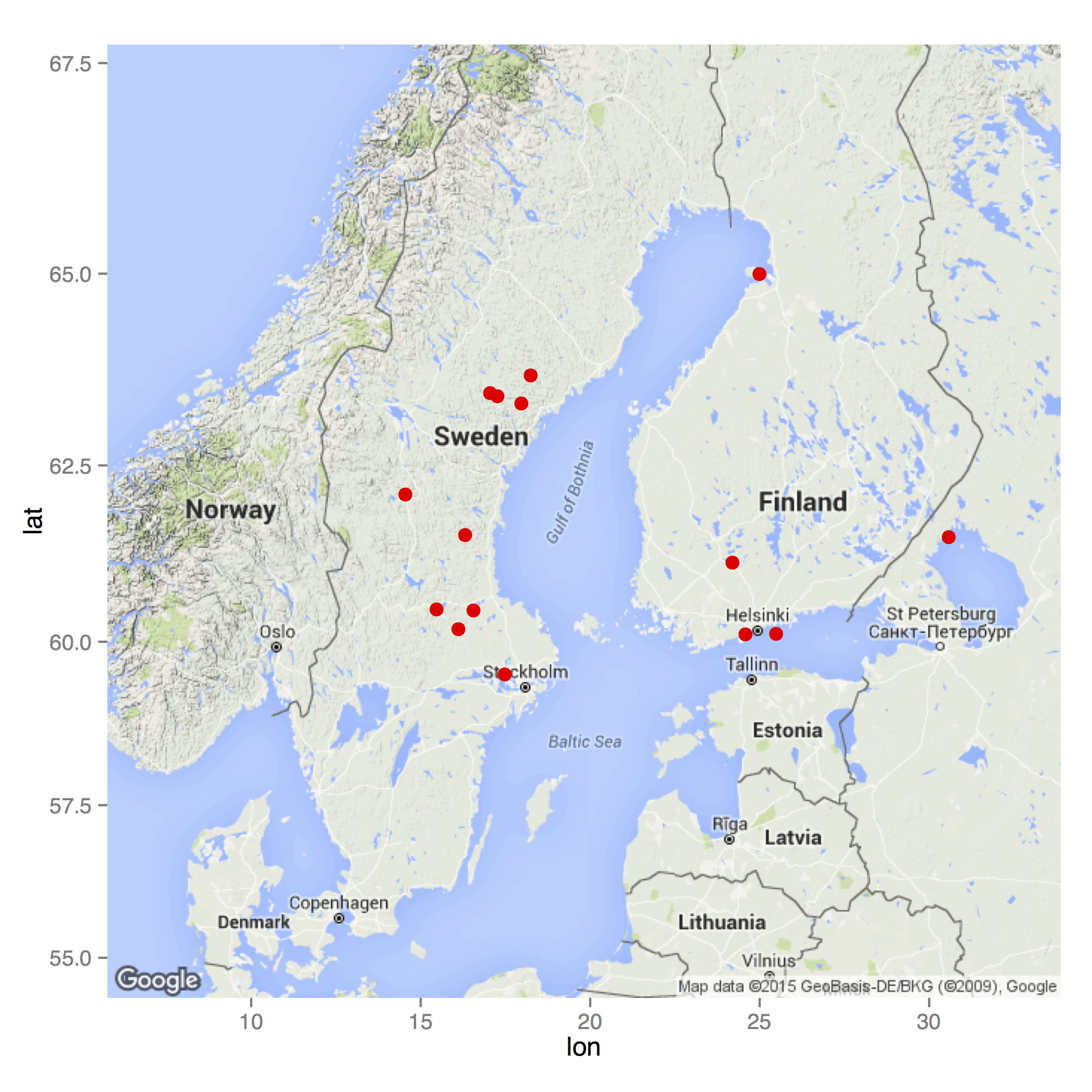


**Fig. S1.** Sampling locations of the 15 analyzed *A. suecica* accessions.


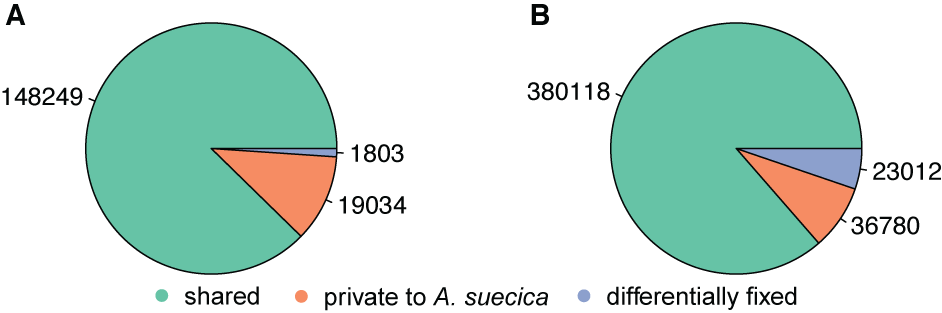


**Fig. S2.** The amount of shared SNPs (in green) between *A. suecica* and *A. thaliana* (**A**) and *A. arenosa* (**B**) contradicts the idea of a single hybridization event giving rise to *A. suecica*.


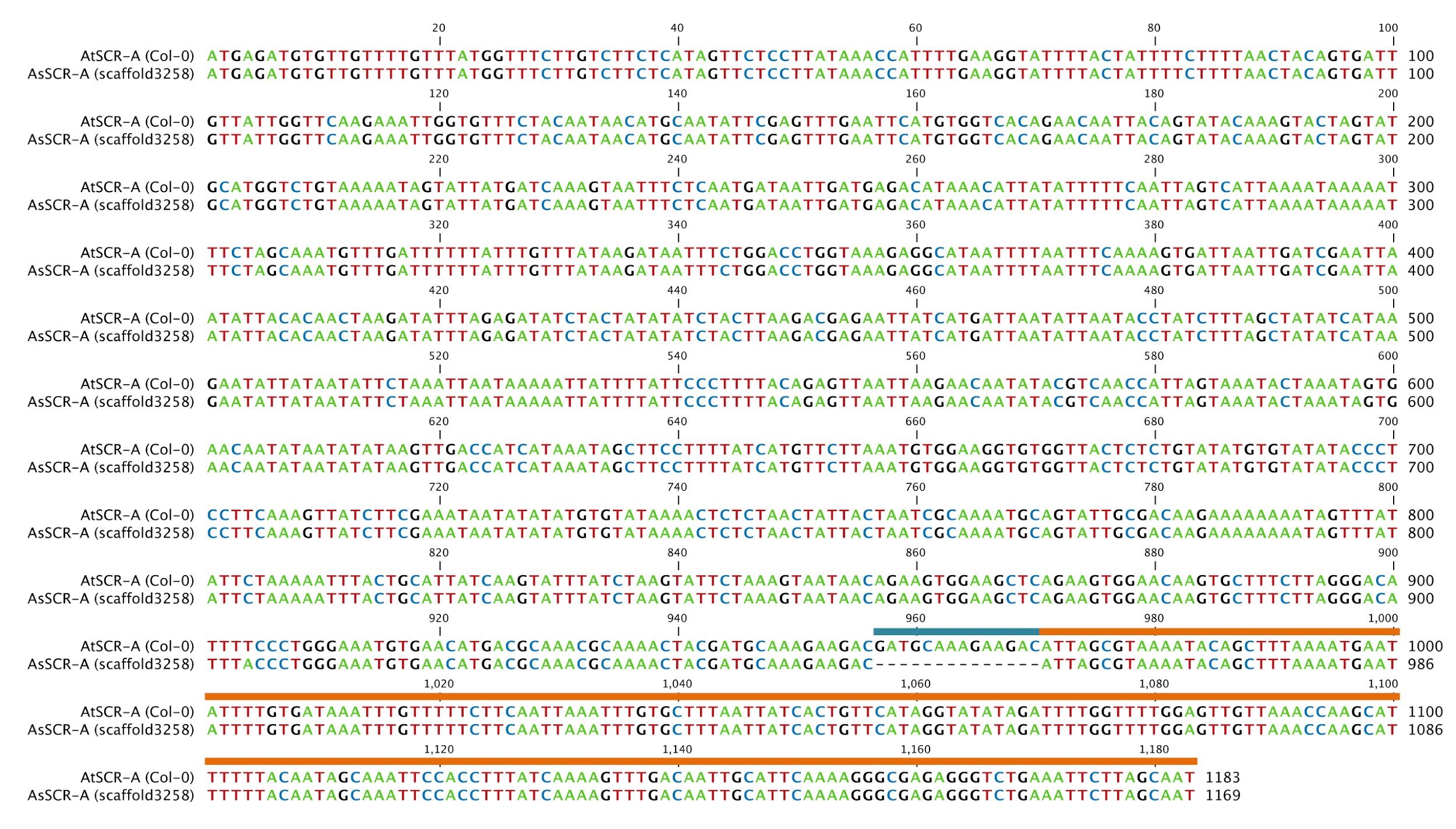


**Fig. S3.** Alignment of the *SCR-A* gene from *A. thaliana* *(Col-0*) and the *A .thaliana*-derived portion of *A. suecica* (scaffold3258). The *A. thaliana* sequence was obtained from the TAIR website (TAIR10 coordinate: Chr4.11382194-11383376; [www.arabidopsis.org/](http://www.arabidopsis.org/)). The alignment starts at the start codon of the *SCR-A* gene and ends with the gene-disruptive 213-bp inversion (Tsuchimatsu, et al. 2010). The 213-bp inversion and the 14-bp duplication are highlighted the by orange and blue lines, respectively. Note that the 14-bp duplication is found in *Col-0* but is not widespread in *A. thaliana* accessions (Tsuchimatsu, et al. 2010).


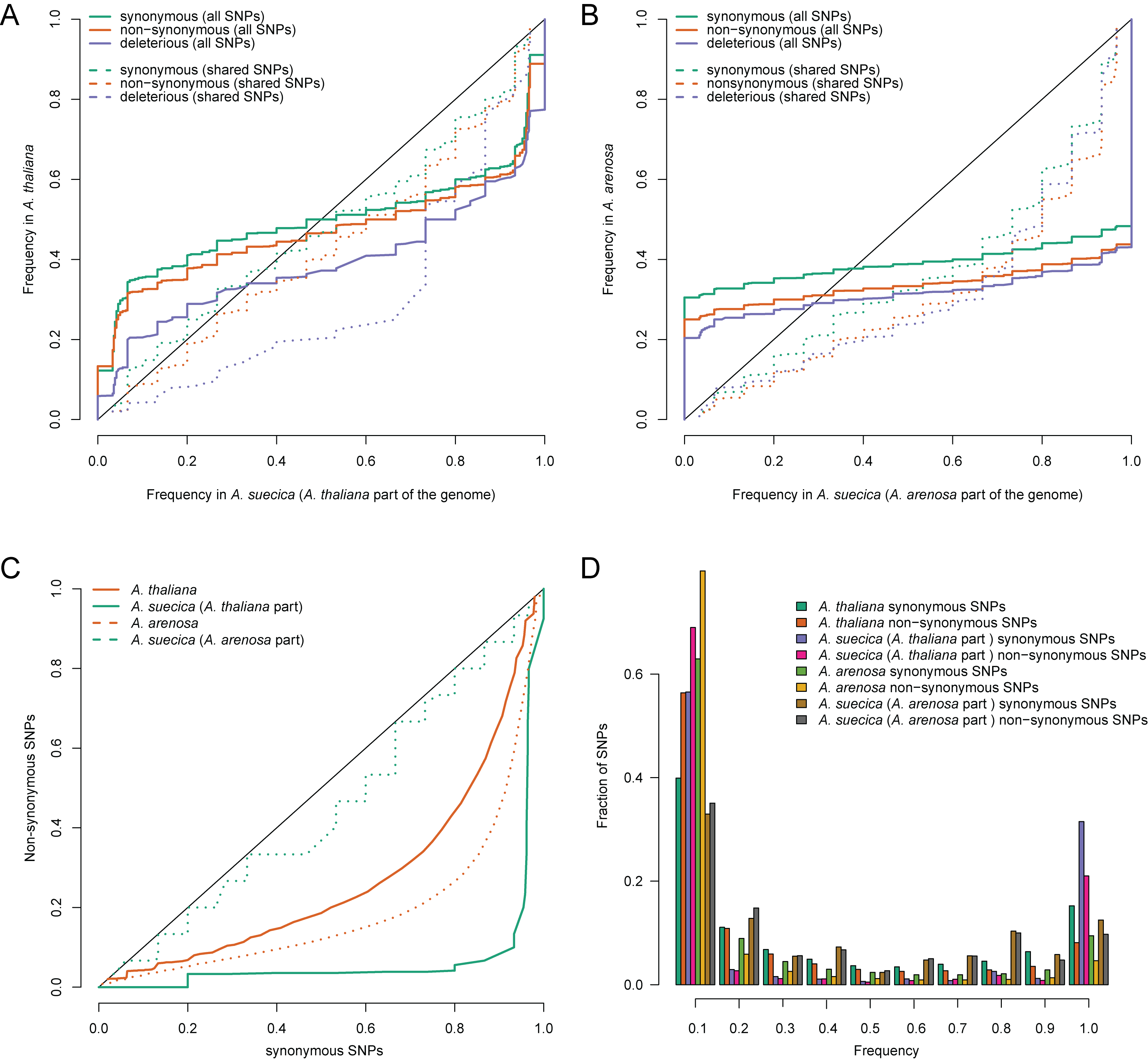


**Fig. S4**. The allele frequency distributions of putatively neutral and putatively deleterious polymorphisms in *A. suecica* and the parental species. (**A**-**B**) Comparison with the parental species demonstrates strong genetic drift presumably due to a bottleneck following hybridization. (**C**-**D**) Analysis of polymorphisms that are private to *A. suecica* demonstrate that purifying selection is acting in the hybrid species. “Deleterious” alleles were identified using SNPeff (Cingolani, et al. 2012) and include stop codons and splice variants that have been gained/lost.


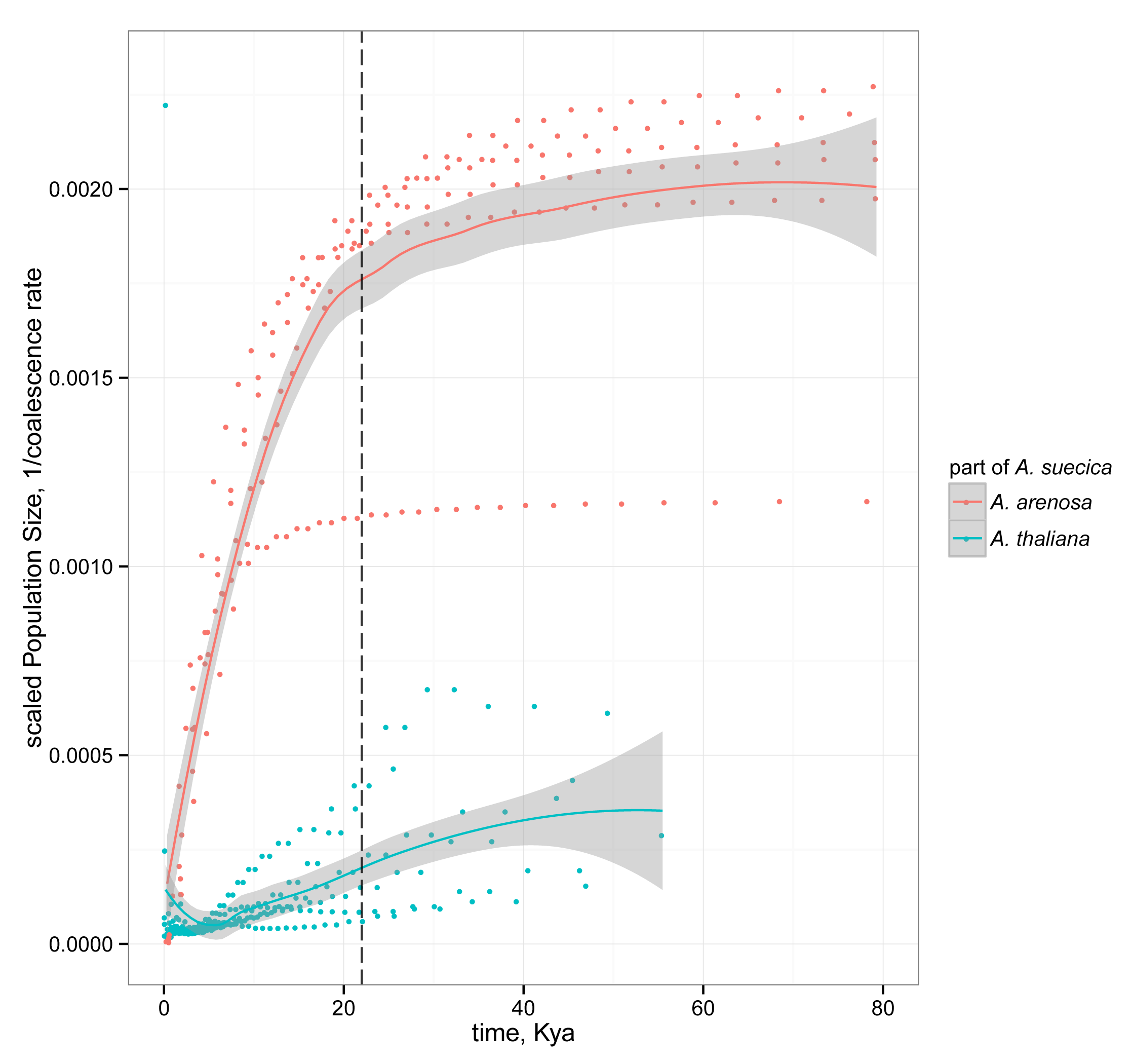


**Fig. S5.** Scaled population size changes over time inferred with MSMC (Schiffels and Durbin 2014) for the *A. thaliana* and *A. arenosa* portions of *A. suecica* (see *Materials and Methods*). Dots represent inferred population sizes at a particular time in the past for a given set of *A. suecica* accessions, while red and blue solid lines represent smoothed means with 0.95 confidence intervals (indicated by the grey area). Population size inferred from both portions of the genome decline following the last glacial maximum (black dashed line at 22 Kya) consistent with the bottleneck associated with *A. suecica's* origin.


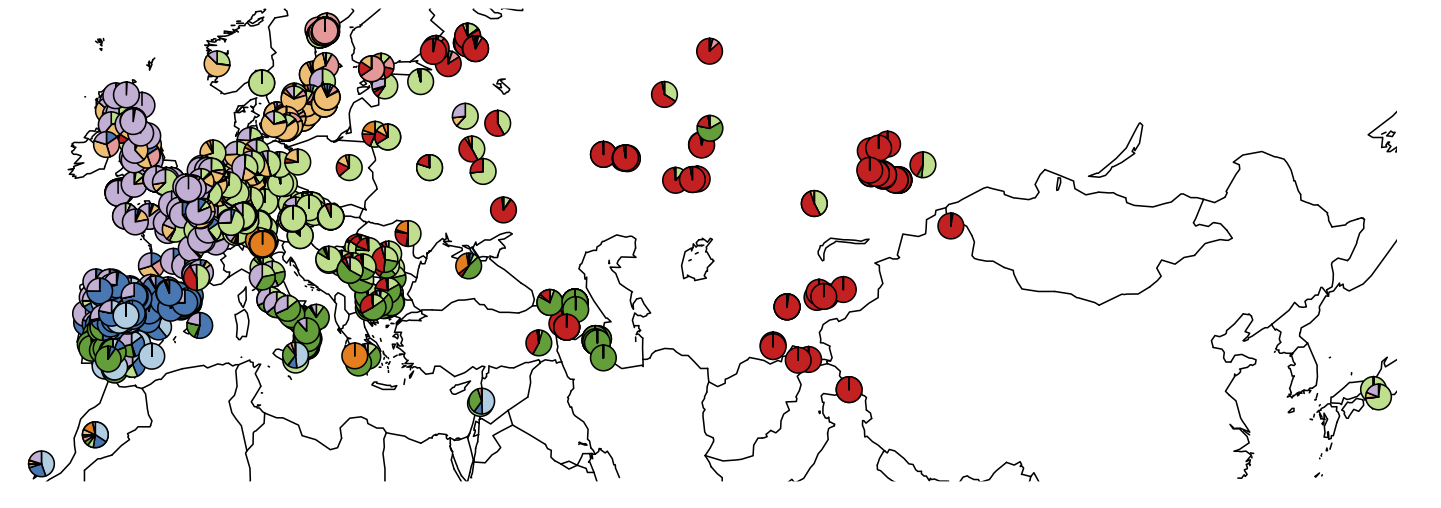


**Fig. S6.** The geographical distribution of the *A. thaliana* ancestral population (in red) for *A. suecica*, based on ADMIXTURE, with K=9 (Alexander, Novembre, and Lange 2009). All *A. suecica* accessions (not shown on this map) are assigned to the red cluster. *A. thaliana* accessions are depicted by pie charts that represent maximum likelihood assignments of individual ancestries, and these are marked by different colors.


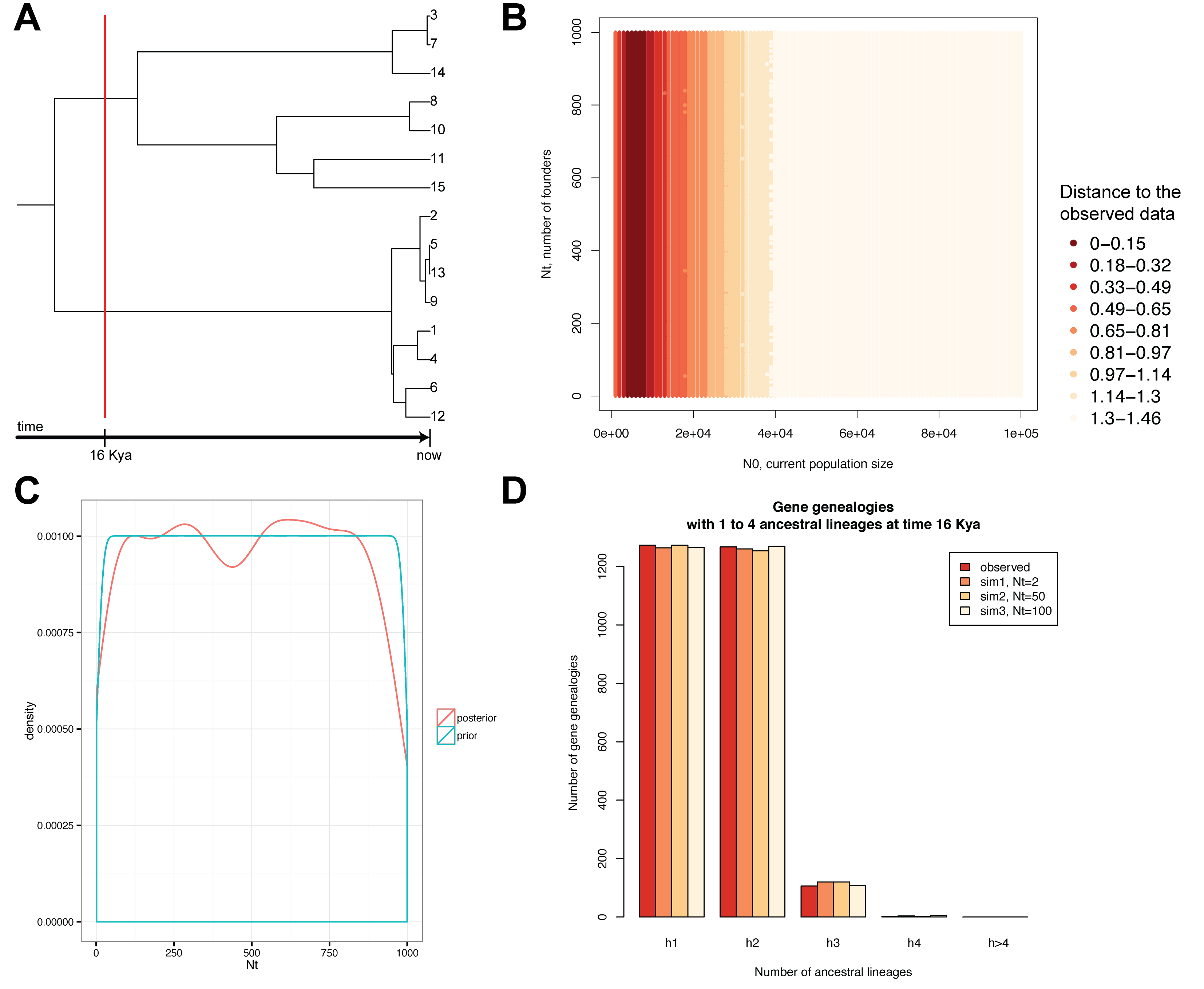


**Fig. S7.** The observed distribution of founder lineages is compatible with a wide range for the number of founders. (**A**) An example of a simulated gene genealogy with 2 ancestral lineages at the origin time of *A. suecica* - 16 Kya. (**B**) The distance to the observed data largely depends on the current population size (minimum distance at N_0_=5,000), but not on the number of founders (ancestral lineages at time 16 Kya). (**C**) Prior and posterior distributions for the number of founders (N_t_ parameter with N_0_ fixed at 5,000). The simulations that are closest to the observed data, with a 0.001 tolerance, were chosen to infer the posterior distribution of the N_t_ parameter. (**D**) The observed counts for the number of gene genealogies with a specific ancestral lineages at 16 Kya, compared with 3 of the simulations: varying the number of founders gives a similar count of gene genealogies.


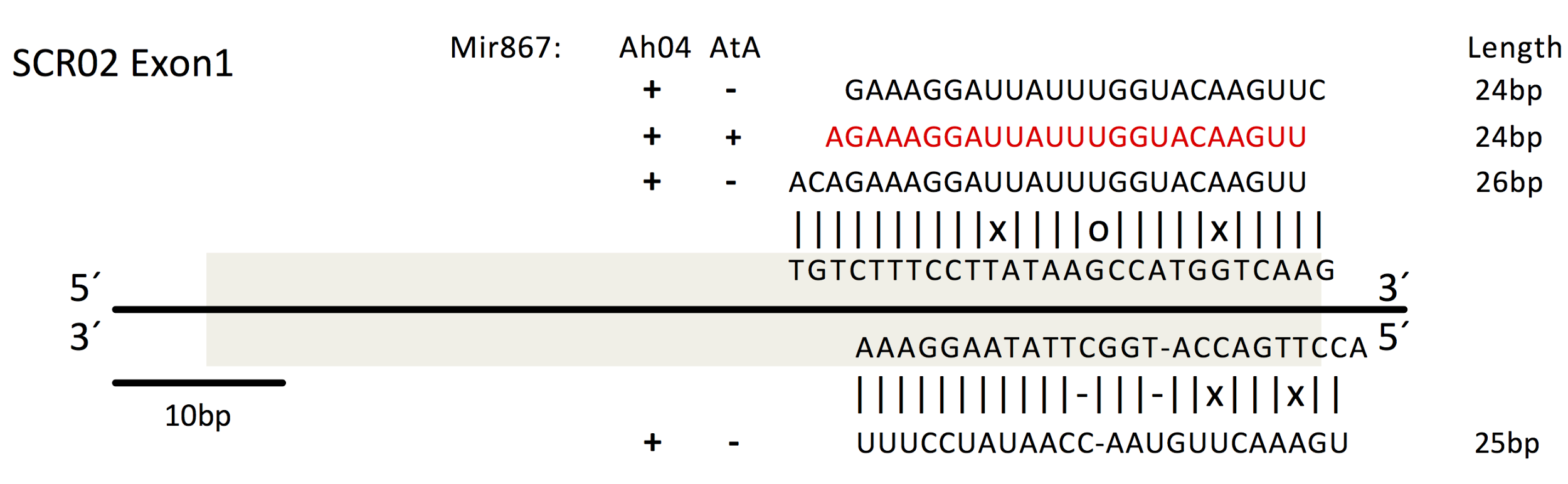


**Fig. S8.** Mir867 expressed in the *A. halleri* S-locus haplotype 4 (Ah04 mir867) and the *A. thaliana* S-locus haplotype A (AtA mir867) is able to target the first exon of *A. halleri*/*A. suecica* SCR02 (denoted by the grey box). Four different small RNAs from the S-locus Ah04 target SCR02, one of which is conserved with mir867 from AtA (highlighted in red).


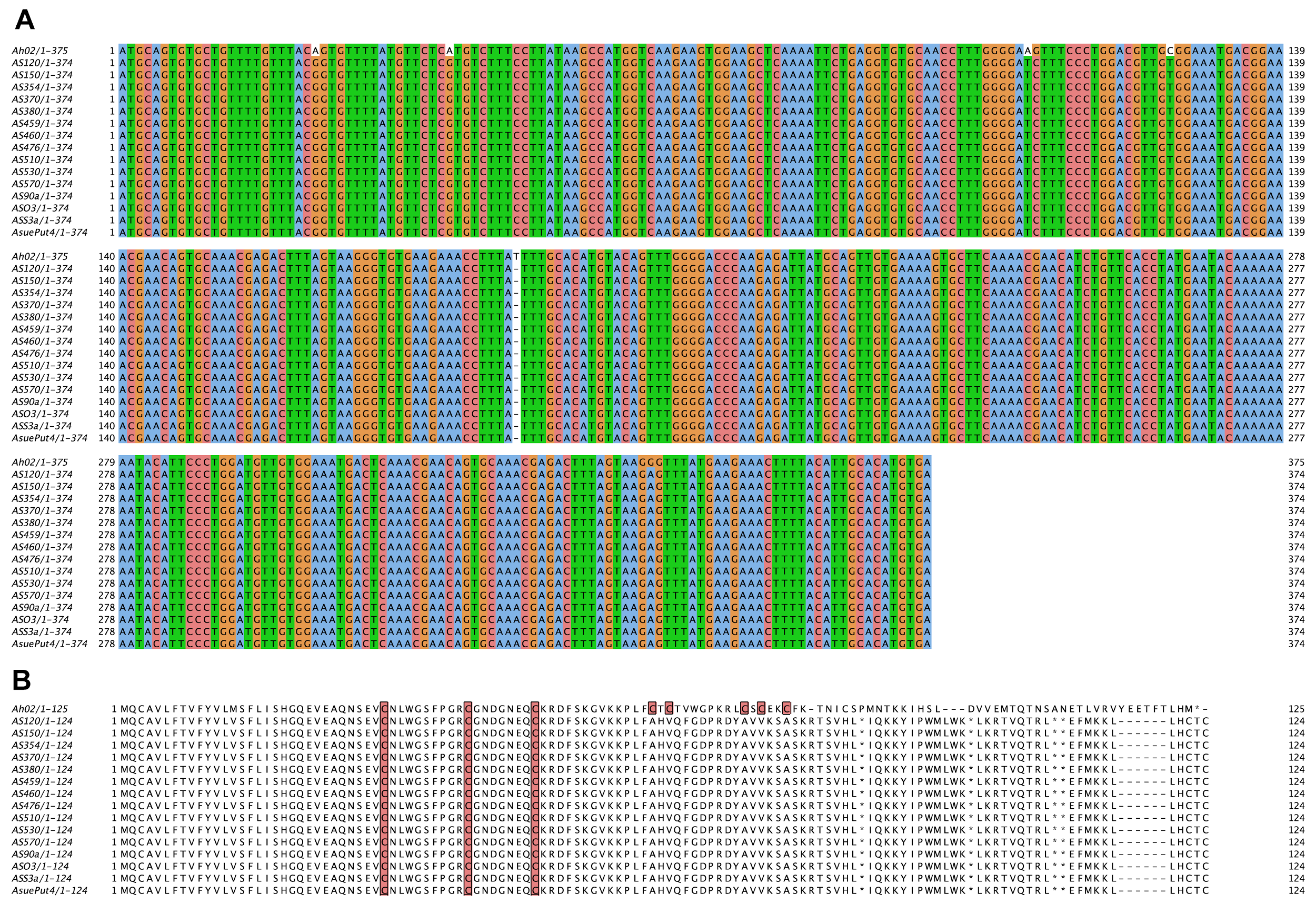


**Fig. S9.** Multiple sequence alignment of *SCR* from *A. halleri* (Ah02 S-haplogroup orthologous to *A. arenosa*-derived S-haplogroup in *A. suecica*) and *A. suecica* (consensus sequence from mapping to Ah02) at the DNA (**A**) and protein (**B**) level. The *A. arenosa*-derived *SCR* gene is likely to be non-functional in *A. suecica*, because it contains a frameshift mutation fixed in all *A. suecica* accessions compared to a functional *A. halleri* *SCR* gene in the orthologous S-haplogroup. The frameshift leads to the loss of 5 out of 8 conserved cysteines (indicated with red color on supplementary fig. S9B) that are important for protein structure (Mishima, et al. 2003; Tsuchimatsu, et al. 2010).


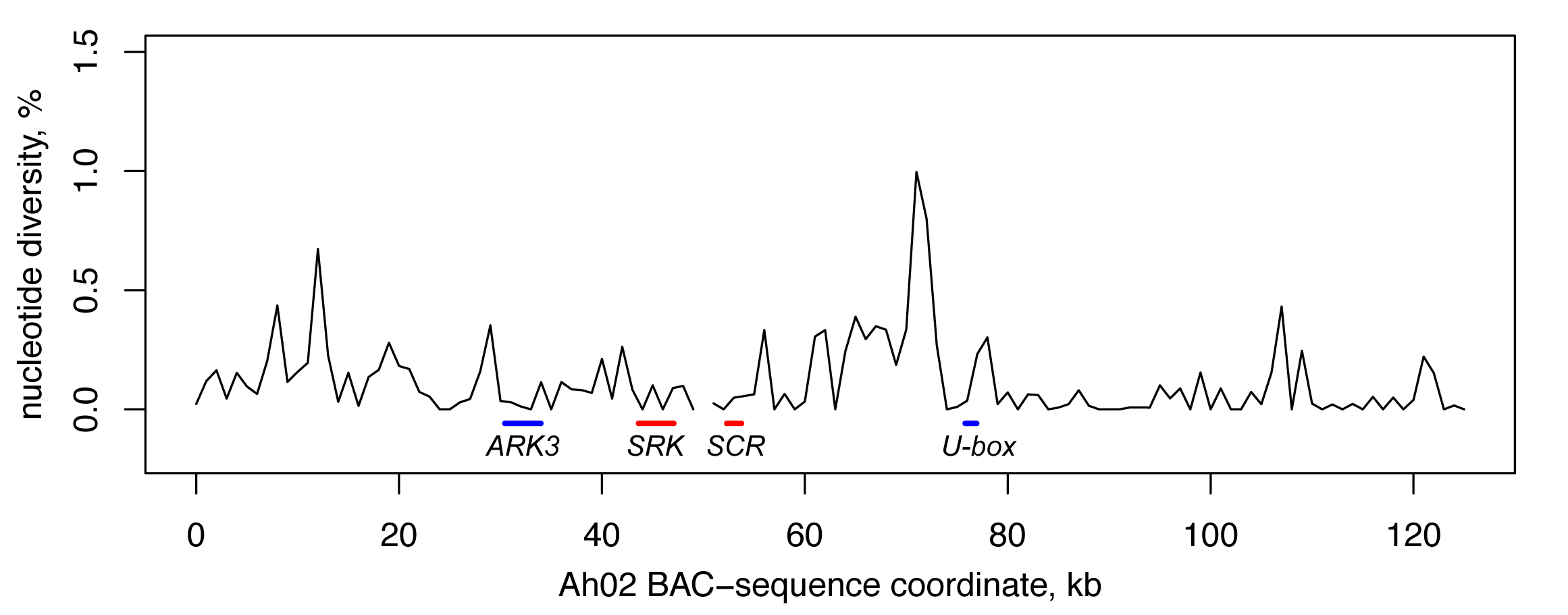


**Fig. S10.** *A. suecica* nucleotide diversity along Ah02 BAC-sequence, calculated in 1-kb windows.


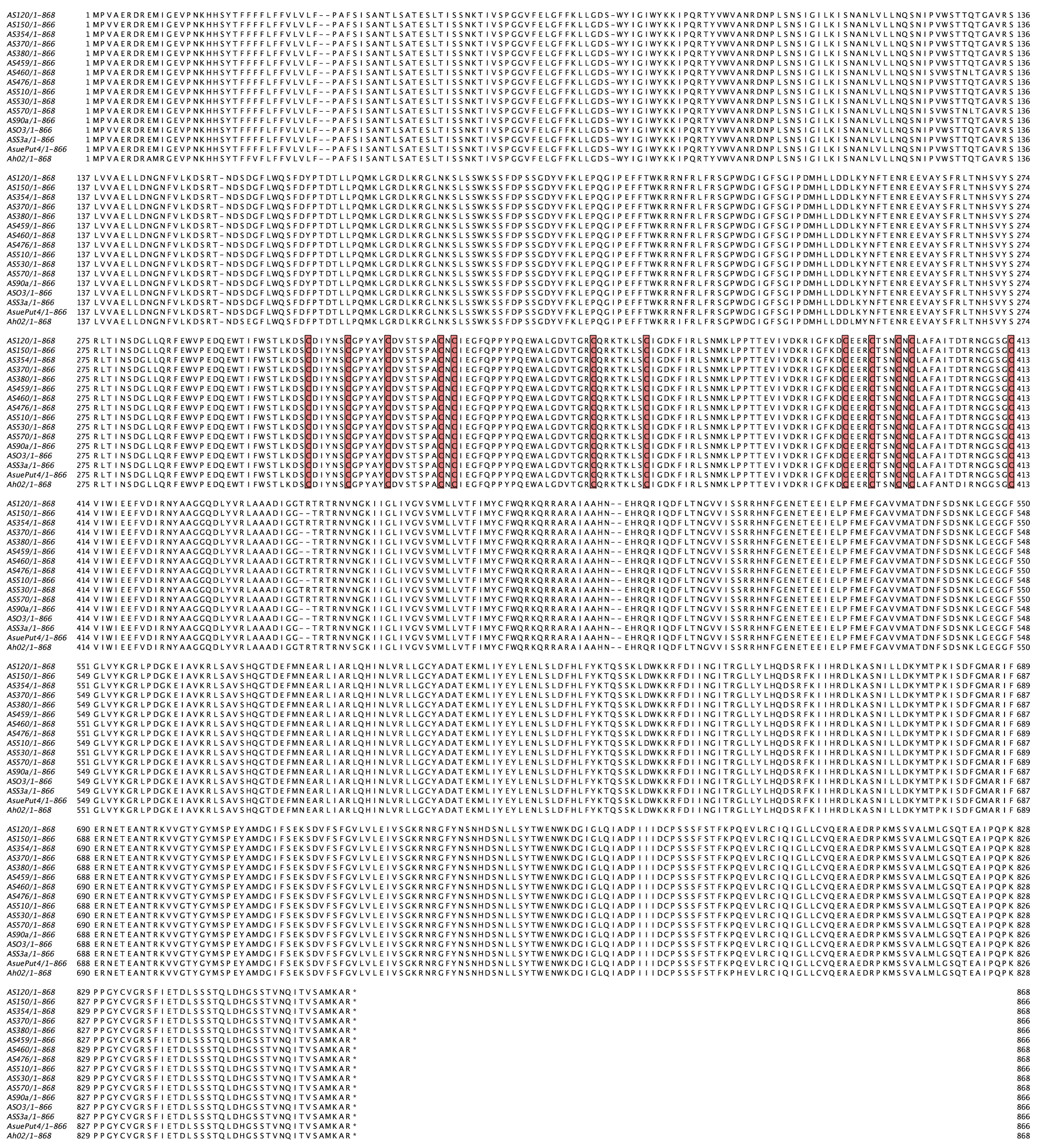


**Fig. S11.** Multiple alignment of predicted amino acid *SRK* sequence from *A. suecica* and *A. halleri* (Ah02 S-haplogroup). The red color indicates the 12 conserved cysteines that have been suggested to be important for the structure of the protein (Kusaba, et al. 1997; Naithani, et al. 2007; Tsuchimatsu, et al. 2012).

**References**

Kusaba M, Nishio T, Satta Y, Hinata K, Ockendon D. 1997. Striking sequence similarity in inter- and intra-specific comparisons of class I SLG alleles from Brassica oleracea and Brassica campestris: implications for the evolution and recognition mechanism. Proc Natl Acad Sci U S A 94:7673-7678.

Naithani S, Chookajorn T, Ripoll DR, Nasrallah JB. 2007. Structural modules for receptor dimerization in the S-locus receptor kinase extracellular domain. Proc Natl Acad Sci U S A 104:12211-12216.

Mishima M, Takayama S, Sasaki K, Jee JG, Kojima C, Isogai A, Shirakawa M. 2003. Structure of the male determinant factor for Brassica self-incompatibility. J Biol Chem 278:36389-36395.
